# Supplementary figures and images for: Leading causes of death and high mortality rates in an HIV endemic setting (Kisumu county, Kenya, 2019)
Source: PLoS One. 2022 Jan 20;17(1):e0261162. doi: 10.1371/journal.pone.0261162 (PMC8775329; doi:10.1371/journal.pone.0261162)

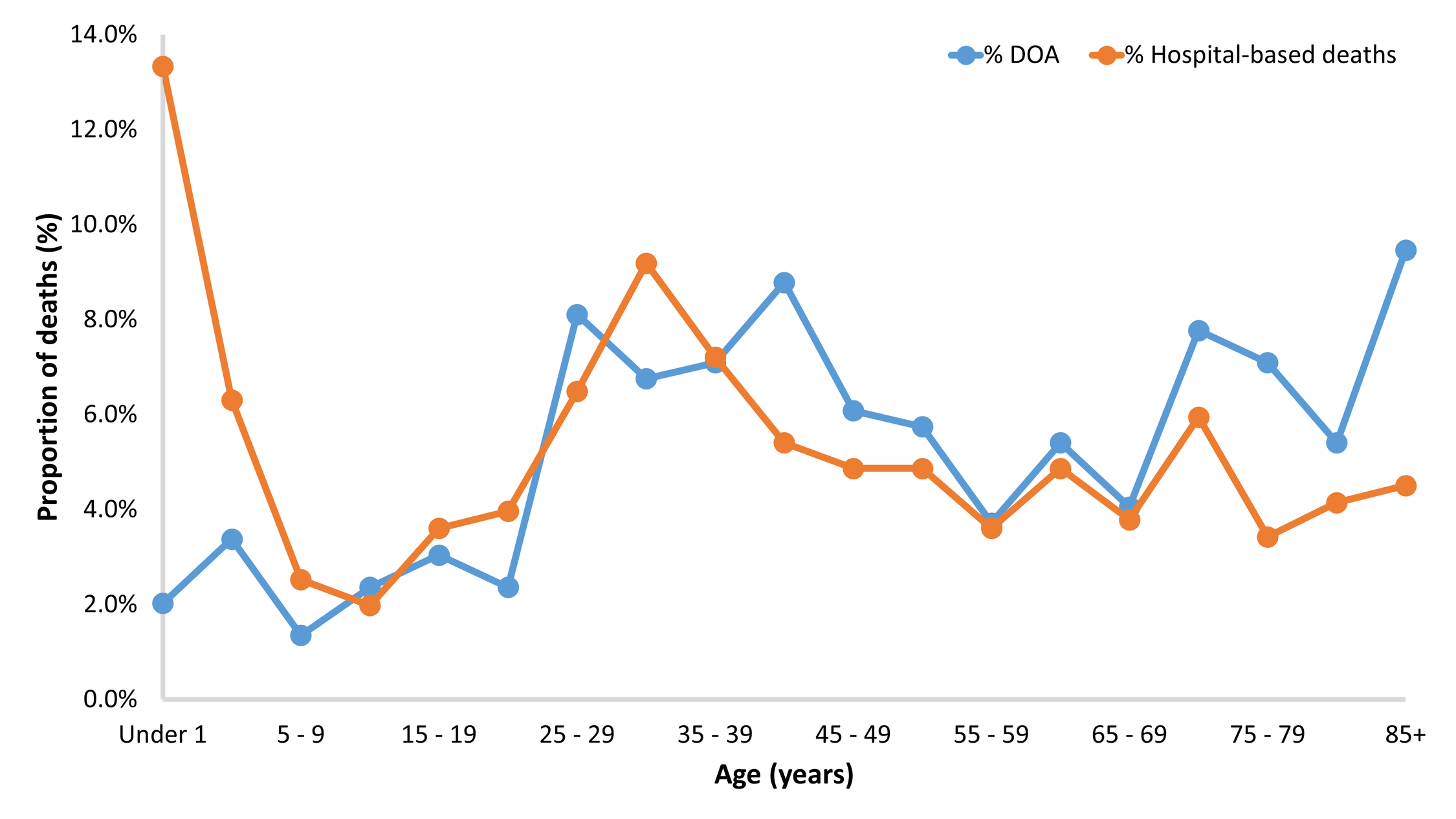

Supplement: S1 Fig — The hospital-based deaths had a higher proportion of children aged <5 years compared to dead on arrival (DOA) cadavers. Age distribution was similar for the rest of the cadavers, except for the DOA group, which had proportionately more cadavers aged ≥70 years than the hospital-based group. (TIF) [file pone.0261162.s001.tif]
