# Supplementary material for: Leading causes of death and high mortality rates in an HIV endemic setting (Kisumu county, Kenya, 2019)
Source: PLoS One. 2022 Jan 20;17(1):e0261162. doi: 10.1371/journal.pone.0261162 (PMC8775329; doi:10.1371/journal.pone.0261162)
Supplement: Attachment — Submitted filename: Response to Reviewers.docx [file pone.0261162.s002.docx]

Friday, June 18, 2021

Editors-in-Chief, PLOS ONE

Dear editors,

Thank you for the review of our manuscript, “**Leading causes of death and high mortality rates in an HIV endemic setting (Kisumu county, Kenya, 2019)**” for publication in *PLOS ONE.* Thank you also for the positive feedback.

We have considered the editorial comments raised, and we have reviewed the manuscript to reflect the requested changes. We have also modified the text further where necessary to improve on clarity. For example, by changing heading levels where appropriate. While reviewing table 5, we realized an anomaly in the rates for HIV-associated and deaths due to HIV/AIDS (they were swopped for the age dissagerations). We have corrected that anomaly. There are no other changes in the contents of the originally submitted manuscript unless otherwise stated above.

There is no change in the financial disclosure statement. Additionally, we have reviewed the reference list to ensure its completeness and correctness. As a result, we have not retracted any references. References [20](https://apps.who.int/iris/bitstream/handle/10665/246208/9789241549165-V3-eng.pdf;jsessionid=211F936CB8E49C27B5CAC3A3152E4E09?sequence=3) and 24 are published documents.

There are no ethical or legal restrictions on sharing a de-identified dataset. Therefore, we have uploaded the de-identified data file in this location <<https://figshare.com/s/e14b72ff064d7bc5467a>>, DOI: [10.6084/m9.figshare.14806176](https://doi.org/10.6084/m9.figshare.14806176).

We hope our paper will receive favorable consideration for publication.

Yours sincerely,

Anthony Waruru, for co-authors
