# Supplementary material for: Leading causes of death and high mortality rates in an HIV endemic setting (Kisumu county, Kenya, 2019)
Source: PLoS One. 2022 Jan 20;17(1):e0261162. doi: 10.1371/journal.pone.0261162 (PMC8775329; doi:10.1371/journal.pone.0261162)
Supplement: Attachment — Submitted filename: Response to Reviewers.docx [file pone.0261162.s003.docx]

Monday, October 25, 2021

Editors-in-Chief, PLOS ONE

Thank you for the review of our manuscript, “**Leading causes of death and high mortality rates in an HIV endemic setting (Kisumu county, Kenya, 2019)**” for publication in *PLOS ONE.* Thank you also for the positive feedback.

We have addressed the specific comments as follows:

**Reviewer #1**:

*Comments*:	* statistical analysis was well prepared in this study
- it is interesting to have this conclusion, it gives a good matching between the background and its results.
- it's a well-written article by following a basic scientific English.
* I recommend to be published.

*Response*: Thank you for the recommendation and taking time to review our manuscript.

**Reviewer #2**:

*Comments*:	Thank you for letting me revise such an interesting paper. It looks like an important work almost ready to be published.
The manuscript well describes the efforts and the work in well detailed & organized way.

Few comments to strengthen its impact are readable here below.

Comment #1. The text of the article has few grammar and typographic errors. The language of the manuscript better to be revised
Comment #2. It is uncommon to join Methods and finding together (Line 44)
Comment #3. It would be good to point to the burden of HIV with recent numbers (Line 132)
Comment #4. Inclusion and exclusion criteria need to be well defined and described
Comment #5. Although main finding well described, I wonder if other than descriptive conducted, need more elaboration

*Responses*:

Comment #1: We have revised the text and corrected grammar and typographic errors.

Comment #2: We have separated the headings

Comment #3. We have separated the two points (population and HIV burden and provided the appropriate reference for each within the sentence)

Comment #4. We have separated the text under ‘study design and population’ and added a new section on ‘inclusion and exclusion criteria’.

Comment #5. The reviewer notes that our analysis is largely descriptive in nature, and appears to be requesting additional analyses beyond those presented, though it is not clear from the comment which specific results the reviewer is requesting. We note that the findings presented are consistent with the study design, whose objectives were to ascertain UCOD, antecedent COD, and immediate COD for hospital-based deaths that occurred in two high-volume referral hospitals in Kisumu County, Kenya, and compare them to those notified to the civil registry, and finally to estimate mortality rates, hence we feel that the current scope and level of detail of the findings are appropriate given the objectives of the study.

We thank the reviewers again and hope that our paper will receive favorable consideration for publication.

Yours sincerely,

Anthony Waruru, for co-authors
